# Hot-Casting Large-Grain Perovskite Film for Efficient Solar Cells: Film Formation and Device Performance

**DOI:** 10.1007/s40820-020-00494-2

**Published:** 2020-07-31

**Authors:** Kejun Liao, Chengbo Li, Lisha Xie, Yuan Yuan, Shurong Wang, Zhiyuan Cao, Liming Ding, Feng Hao

**Affiliations:** 1 School of Materials and Energy, University of Electronic Science and Technology of China, Chengdu, 610054 People’s Republic of China; 2 Center for Excellence in Nanoscience (CAS), Key Laboratory of Nanosystem and Hierarchical Fabrication (CAS), National Center for Nanoscience and Technology, Beijing, 100190 People’s Republic of China

**Keywords:** Perovskite film, Hot-casting, Temperature, Precursor chemistry, Grain size

## Abstract

Organic–inorganic metal halide perovskite solar cells (PSCs) have recently been considered as one of the most competitive contenders to commercial silicon solar cells in the photovoltaic field. The deposition process of a perovskite film is one of the most critical factors affecting the quality of the film formation and the photovoltaic performance. A hot-casting technique has been widely implemented to deposit high-quality perovskite films with large grain size, uniform thickness, and preferred crystalline orientation. In this review, we first review the classical nucleation and crystal growth theory and discuss those factors affecting the hot-casted perovskite film formation. Meanwhile, the effects of the deposition parameters such as temperature, thermal annealing, precursor chemistry, and atmosphere on the preparation of high-quality perovskite films and high-efficiency PSC devices are comprehensively discussed. The excellent stability of hot-casted perovskite films and integration with scalable deposition technology are conducive to the commercialization of PSCs. Finally, some open questions and future perspectives on the maturity of this technology toward the upscaling deposition of perovskite film for related optoelectronic devices are presented.

## Highlights

- Recent advances of a hot-casting technique used to deposit high-quality perovskite films are reviewed.
- Perovskite films with large grain size, uniform thickness, and preferred crystalline orientation are deposited.
- Future perspectives on the upscaling of perovskite solar cell are described.

## Graphical abstract

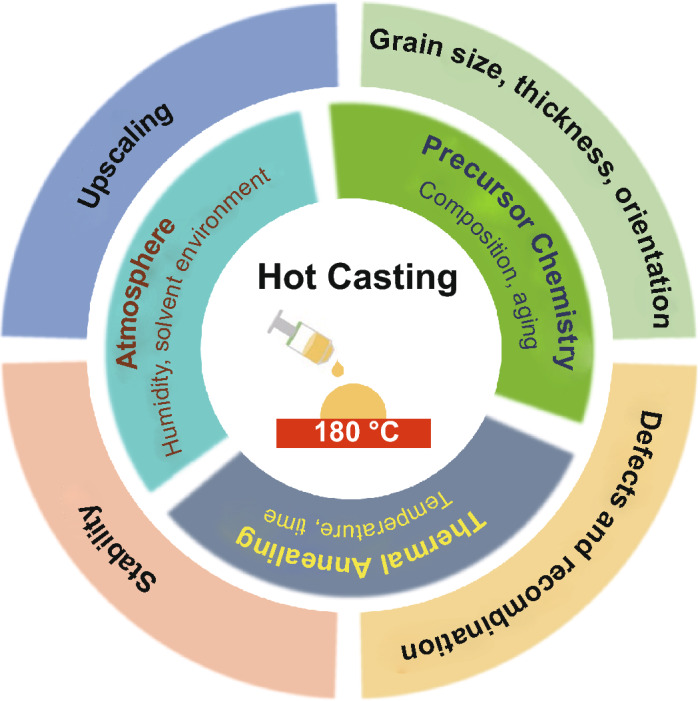

## Introduction

The development of low-cost and high-efficiency solar cells has attracted interest worldwide from both the academy and industry. Organic–inorganic lead halide perovskite has recently emerged as an efficient light-absorbing layer in photovoltaic applications with unique properties including a high absorption coefficient, tunable bandgap, and solution processing capability [1–8]. The improvement in the power conversion efficiency (PCE) of perovskite solar cells (PSCs) is mainly due to the effective control of the film morphology and the interfacial defect passivation. Therefore, the choice of deposition technique is particularly important. One-step spin coating [9–12], sequential deposition [13], two-step spin coating [14, 15], and vapor deposition [2, 16] have been widely used to deposit high-quality perovskite films. Despite being fairly complicated, sequential deposition and two-step spin coating exhibit good repeatability and high controllability. Vapor deposition is suitable for planar devices with a high film uniformity, although deposition under a strong vacuum is expensive. Crystallization in a conventional one-step spin coating is difficult to control because the deposition environment such as the humidity and temperature seriously affects the repeatability, and the thermal annealing process involves evaporation of the solvent and volatilization of the organic components.

Hot-casting technology has recently been developed in the deposition of high-quality perovskite thin films with notable advantages, such as a rapid crystallization, short film formation process, large increase in the grain size, preferred crystalline orientation, and low defect states [17–19]. A direct formation mechanism has been proposed within the framework of nucleation growth theory [20]. A high substrate temperature provides the necessary driving force for the phase change, resulting in an ultrashort crystallization process. Meanwhile, sufficient thermal energy also facilitates the diffusion of atoms in a liquid without the formation of an intermediate phase. Nie et al. first reported this methodology and further improved the film quality by optimizing the deposition parameters, such as the substrate temperature [19, 21], annealing temperature [22], and precursor composition [11]. This technology has recently been extended to the deposition of a variety of perovskite films, including organic–inorganic hybrid, all-inorganic, lead-free, and low-dimensional perovskite films [23–25].

In this review, the recent developments and advances of the hot-casting deposition of perovskite films are comprehensively summarized and discussed from the perspective of film quality, defects, carrier recombination, and stability. First, classic nucleation and crystal growth theory are revisited. The effects of different deposition parameters in a hot-casting technique, including the substrate temperature, thermal annealing, precursor chemistry, and atmosphere, are further discussed. Next, the advantages of this promising technology are discussed in terms of grain size, uniform thickness, and preferred crystalline orientation. The implementation of this technology in different types of perovskite film deposition has also been reviewed. Finally, some open questions and future perspectives regarding the maturity of this technology toward the upscaled manufacturing of perovskite-related optoelectronic devices are presented.

## Hot-Casting Technology

### Fundamentals of Nucleation and Crystal Growth

**Fig. 1:**
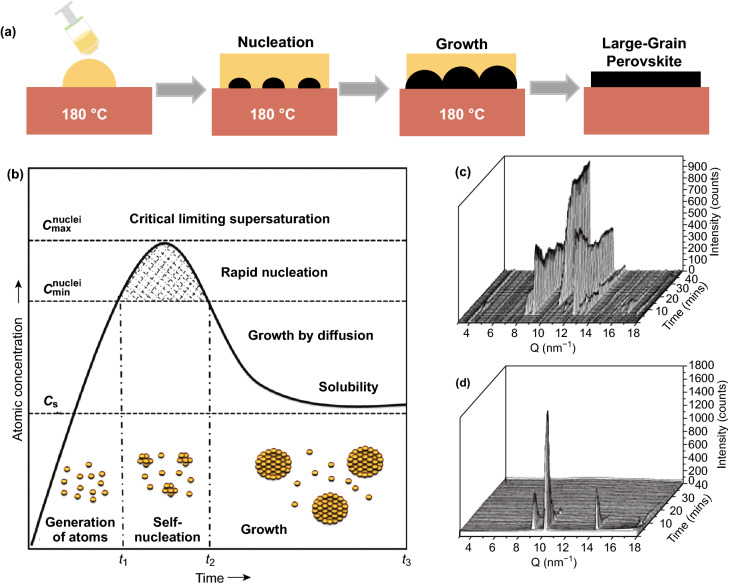
**a** Schematic illustration of the hot-casting process. **b** LaMer diagram for crystallite formation stages in a solution [29]. Copyright © 1950 American Chemical Society. **c** Time-dependent grazing-incidence wide-angle X-ray scattering (GIWAXS) profiles of the perovskite film drop cast on the 70 °C substrate and **d** 180 °C substrate from a solution to a solid state (0–40 min) [20]. Copyright © 2016 American Chemical Society

Classic nucleation and the crystal growth mechanism are first revisited [26–28]. Figure 1a describes a schematic illustration of a hot-casting process for perovskite film deposition. The hot-casting technology aims to spin coat a hot precursor solution on a substrate maintained at higher temperature. Factors such as the substrate temperature, solution concentration, solvent, and supersaturated environment affect crystal growth. These deposition parameters are comprehensively discussed and correlated with the processes of nucleation and growth, as indicated in a LaMer diagram.

#### Classical Nucleation and Classical Growth

There are three classic nucleation and growth models of thin films, including the Volmer–Weber model, Frank–van der Merwe model, and Stranski–Krastanov model [30–33]. For the nucleation and growth of most polycrystalline thin films, the Volmer–Weber model is valid if the substrate temperature is sufficiently high and the deposited atoms have a certain diffusion capability. Therefore, our discussion is based on the Volmer–Weber model for hot casting process with unique film-forming characteristics. In classical nucleation theory, the solution must be in a supersaturated state to initiate crystallization. Nucleation with preferential nucleation sites is called a heterogeneous nucleation, which means that new phases are preferentially formed within certain regions of a liquid phase. During the process of thermodynamic nucleation, the system needs to overcome an energy barrier, which is the maximum free energy of critical nucleation ($\Delta G^{*}$).

1 $$\Delta G^{*} = \frac{{16\pi \sigma^{3} }}{{3\Delta G_{v} }}$$

where $\sigma$ is the interface free energy, and $\Delta G_{v}$ is the bulk free energy. Formation of nuclei in solution is closely related to the critical radius (r*):

2 $$r^{*} = \frac{2\sigma }{{\Delta G_{v} }}$$

when *r* < r*, the crystal nucleus is unstable; when *r* = r*, the crystal nucleus is in a metastable state; when *r* > r*, it is converted into a stable crystal nucleus and large nucleation begins. Therefore, r* represents a critical value for the transition from an unstable crystal nucleus to a stable crystal nucleus, i.e., r* represents the minimum size at which the particles are further grown in a solution without being dissolved.

#### LaMer Mechanism

According to the typical concentration distribution curve in the LaMer diagram shown in Fig. 1b, the process of nucleation and growth can be divided into three stages, i.e., regions I, II, and III, representing the prenucleation, nucleation, and growth stages, respectively. In LaMer mode, the level of atomic concentration is the direct control factor for generating the crystal nuclei and achieving the particle size growth. Here, *C*_s_ is the solubility limit of the solution, *C*_c_ is the critical concentration of the solution, and *C*_m_ is the maximum supersaturation. In the 0–*t*_1_ segment, the centrifugal force and substrate temperature promote the solvent evaporation and increase the solution concentration of precursor until reaching *C*_s_, although no obvious nucleation occurs at this point. The deposition temperature, precursor solution concentration, deposition rate, and solvent composition will affect the prenucleation stage. In the *t*_1_–*t*_2_ segment, the concentration of the solution reaches the critical level of nucleation, and the nucleation radius is larger than r*. The nucleation is transformed into a stable crystal nucleus, and a stable heterogeneous nucleus is formed at the beginning of slow nucleation. After the precursor solution is dropped onto the substrate, when the binding energy between the crystal nucleus and the adsorption atom is higher than the binding energy between the adsorption atom and the substrate, the crystal nucleus is more likely to form islands in combination with the diffusion-migrating atom. During the nucleation stage of the hot-casting process, a high substrate temperature, a diffusion capacity of the deposited atoms, and wettability between the deposited material and the substrate are essential requirements. Simultaneously, the precursor chemistry such as the solution aging time, solution composition, and solvent selection are important factors. The nucleation rate in *t*_2_–*t*_3_ segment is rapidly increasing. Secondary nucleation occurs in an irregular channel. The distance between the islands gradually decreases and merges into a large island or forms a continuous film. At this stage, the solution concentration is closely related to the nucleation and growth rate. The balance of the nucleation and growth rate effectively improves the film coverage [34]. When the solution concentration is below the critical supersaturation, nucleation stops owing to a low concentration of the solution [35]. The thermal annealing facilitate a fine control of the volatilization of the components and solvents, and ultimately a high-quality perovskite film is obtained.

### Temperature and Thermal Annealing

#### Direct Formation Mechanism

The substrate temperature and thermal annealing play crucial roles in nucleation growth. When the substrate temperature is relatively low (less than 100 °C), the perovskite film formation process is divided into three stages: the initial solution stage, the transition-to-solid film stage, and the transformation stage from intermediates into a perovskite film [36]. However, when the substrate temperature is increased (100–180 °C), the formation of the perovskite film adopts a direct formation mechanism [20]. Figure 1c, d shows in situ grazing-incidence wide-angle X-ray scattering (GIWAXS) curves of perovskite from the precursor solution to the perovskite film at different substrate temperatures of 70 and 180 °C, respectively. The X-ray data are acquired after a regular time interval, and the time coordinate (right) represents the characteristic peak obtained within each frame interval. The peak of the scattering vector (*Q*) at ~ 4, 8, and 8.7 nm^−1^ is attributed to the PbI_2_–solvent complex. The peak at *Q* = 12.0 nm^−1^ is assigned to the intermediate-stage MAPbCl_3_–solvent complex, whereas the peak at *Q* = 10.2 nm^−1^ is due to the perovskite stage. The intermediate MAPbCl_3_ and PbI_2_–solvent complex was not seen in the film grown at 180 °C. This indicates that there is no intermediate phase formation during the hot-casting process, since sufficient thermal energy is provided to accelerate the reactant diffusion and interaction.

#### Substrate Temperature

**Fig. 2:**
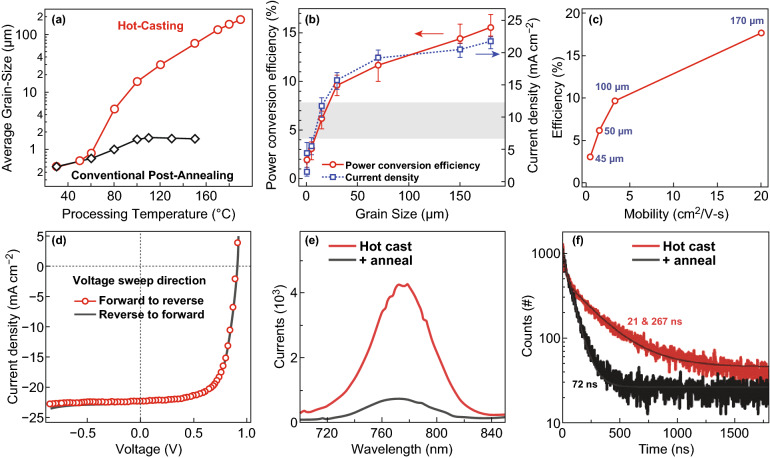
**a** Comparison of grain size as a function of the processing temperature. **b** Average overall PCE (left) and *J*_sc_ (right) as a function of crystalline grain size. **c** PCE values are correlated with the bulk mobility of the perovskite layer (the labels correspond to the average grain size). **d** Average *J*–*V* characteristics resulted by sweeping the voltage from forward to reverse bias and from reverse to forward bias [19]. Copyright © 2017 American Association for the Advancement of Science. **e** PL and **f** TRPL spectra of unannealed and annealed perovskites [22]. Copyright © 2016 WILEY-VCH Verlag GmbH & Co. KGaA, Weinheim

The substrate temperature affects the nucleation rate and film morphology by changing the supersaturation of the solution. Nie et al. first reported a strong correlation between the substrate temperature and grain size during a hot-casting process (Fig. 2a), as well as the mobility and final PCE (Fig. 2b–d). When the substrate temperature is higher than the crystallization temperature of the perovskite phase, the high boiling solvent promotes a stable growth of the perovskite crystal with large crystal grains. When the substrate temperature is increased to 180 °C, a grain size of 180 μm and charge mobility of 20 cm^2^ V^−1^ s^−1^ are obtained in the perovskite film. A large-grain perovskite film improves the device performance in two ways, reducing the crystal interface and lowering the defect density, thus increasing the mobility and suppressing the charge trapping [19].

#### Thermal Annealing

Yang et al. further described the Volmer–Weber growth mechanism for the formation of island-like grains and the transition to a dense perovskite film in a hot-casting process [37]. The thermal energy plays a key role in accelerating the crystallization of perovskite and the diffusion of the precursor, which directly determines the film morphology (Fig. 1b). For instance, the thermal energy can reduce the surface tension between the precursor solution and the substrate, thus enhancing the tendency to form large islands with reduced surface defects (such as vacancies, dislocations, and grain boundaries). As the temperature further increases, it was observed that isolated islands grow and begin to form bonds with each other, eventually forming a high-quality perovskite film without pinholes. Thermal annealing not only promotes the solvent evaporation and accelerates the material transport within the film, but also improves the charge transport in the perovskite film. Janssen et al. characterized the annealing procedure using in situ photoluminescence spectroscopy [22]. The thermal annealing of the perovskite layer on poly(3,4-ethylenedioxythiophene)/poly(styrenesulfonate) (PEDOT:PSS) affects the photoluminescence properties, as shown in Fig. 2e, f. The luminescence intensity after annealing was 7 times lower than that of the as-cast film, and the photoluminescence lifetime (72 ns for a single-index fit) was significantly shorter than that of the as-cast film (double exponential lifetime of 21 and 267 ns). This difference was due to the increased charge carrier mobility or fewer defects in the annealed film. However, if the annealing temperature is too high and the annealing time is too long, it will promote the decomposition of the perovskite and eventually damage the photovoltaic performance. Ma et al. investigated the relationship between the thermal annealing and orientation of hot-casted PEA_2_MA_2_Pb_3_I_10_ films [38]. Nateghi et al. also reported that the changes in annealing temperature and substrate temperature affect the morphology of the deposited film [39]. Flash infrared annealing (FIRA) can promote the formation and crystallization of perovskite films through rapid solvent removal. Compared to conventional thermal annealing, short heating pulses can significantly reduce the degradation of organic components even at an extremely high temperature [40, 41]. Ren et al. found that the grain size distribution at the edge of the perovskite film prepared using hot casting is larger than the central size distribution and the particle size distribution is annular [42]. It is believed that a compensation flow from the center of the solution to the edge naturally occurs during the evaporation of the solvent, thus resulting in a higher concentration and larger grain size at the edges.

### Precursor Chemistry

#### Additives

**Fig. 3:**
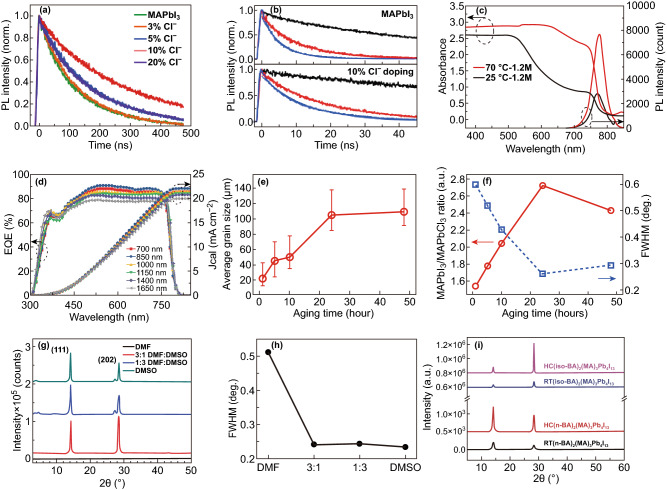
**a** TRPL curves for a pristine perovskite film and with different chlorine contents. **b** TRPL curves for pristine perovskite films (black) in conjunction with a hole quencher (red) and an electron quencher (blue) [11]. Copyright © 2016 WILEY-VCH Verlag GmbH & Co. KGaA, Weinheim. **c** Absorption spectra and steady-state PL emission spectra of MAPbI_3_ perovskite films fabricated at different casting temperatures of 25 °C and 70 °C. **d** EQE spectra for perovskite solar cells with varying active layer thicknesses through hot cast [53]. Copyright © 2016 WILEY-VCH Verlag GmbH & Co. KGaA, Weinheim. **e** Average grain size with error bar determined for 1–48 h aging time. **f** Peak ratio of MAPbI_3_/MAPbCl_3_ as a function of aging time and FWHM of MAPbI_3_ peak (2*θ* = 14.28°) [46]. Copyright © 2017 WILEY-VCH Verlag GmbH & Co. KGaA, Weinheim. **g** XRD patterns as a function of peak intensity versus 2θ (degree) of the hot-cast BA_2_MA_4_Pb_5_I_16_ films from precursor solutions using DMF, DMSO, and mixed solvents. **h** FWHM of the (111) reflection as a function of precursor solvent used for thin-film fabrication [17]. Copyright © 2017 WILEY-VCH Verlag GmbH & Co. KGaA, Weinheim. **i** XRD patterns of RT and hot-cast fabricated (n-BA)_2_(MA)_3_Pb_4_I_13_ and (iso-BA)_2_(MA)_3_Pb_4_I_13_ perovskites [18]. Copyright © 2019 Elsevier Ltd.

The perovskite precursor has a strong impact on the film morphology and device performance, such as the composition [43, 44], concentration [45], solution aging time [46], and solvent selection [47]. A poorly soluble inorganic lead salt requires the selection of strongly polar aprotic organic solvents. The boiling point and vapor pressure of the solvent determine the rate of solution evaporation. The viscosity of the solvent affects the substrate wettability and thus the film formation [48]. In addition, the Lewis-based nature of some high polar aprotic organic solvents can induce a solvent–solute coordination to modulate the crystallization process. Therefore, the solvation ability plays a key role in determining the perovskite crystallization process [49]. The perovskite precursor solution is regarded as a colloidal cluster with a soft colloidal skeleton, and several coordination complexes constitute the colloidal skeleton. The size of the colloidal clusters is controlled by additives (Cl^−^ and Br^−^) [50]. Liao et al. reported that the incorporation of chlorine can improve the optoelectronic properties and environmental stability of perovskite films. The incorporation of 10 wt% Cl^−^ into a MAPbI_3_ precursor solution significantly improves the film uniformity and coverage. An X-ray diffraction (XRD) pattern showed that increasing the chlorine amount induces a significant increase of the scattering intensity of the (110) plane, whereas the intensity of the (020) plane is decreased, indicating a promoted orientation in the direction of the (110) plane. The orientation is highly dependent on the halide distribution during the hot-casting process. The solvent evaporates instantly upon contact with the hot substrates and draws the preaggregated Cl^−^-rich domains toward the surfaces. Cl^−^ preferentially distributes closer to the TiO_2_ substrates, and the included Cl^−^ precursors tend to naturally aggregate owing to a lower solubility. Consequently, the rapid crystallization creates a gradient halide distribution as well as morphological changes. A high nucleation density and grain coarsening are further induced. A longer time-resolved photoluminescence (TRPL) lifetime was observed in a perovskite film with added chloride ions (Fig. 3a, b). Improvements in the device performance have been ascribed to the improved uniformity of the perovskite film, good orientation of the perovskite crystals, and a longer carrier diffusion length. Wu et al. reported that Cl^−^ can improve the film morphology by retarding the crystallization rate, whereas Br^−^ can improve the open-circuit voltage (*V*_oc_) of the corresponding devices and stabilize the perovskite lattice [51]. Hao et al. reported that a slight excess of methylammonium iodide (MAI) in the precursor solution can compensate for the MAI loss owing to the high substrate temperature [52]. Significant increases in the grain size and crystallinity of the MAI-rich perovskite films were observed. Chen et al. further tuned the thickness of the perovskite layer within the range of 700–1600 nm by adjusting the substrate temperature (25–100 °C) and the precursor concentration (1–2 M) [53]. Figure 3c, d shows the UV–Vis absorption and external quantum efficiency (EQE) spectra for the corresponding devices. Within the long-wavelength range, the absorption was enhanced from 25 to 70 °C, thereby contributing to enhanced light-harvesting and improved *J*_sc_ values. The photoluminescence (PL) intensity of a sample at 70 °C is much higher than that at 25 °C, indicating fewer trap states in the former. Gong et al. further used an ultrahigh-temperature substrate (~ 240 °C) to deposit a perovskite film in a short time owing to the rapid evaporation of the solvent [54].

#### Aging Time and Solvent

The aging time of the precursor solution can affect the size and structure of the colloidal clusters, as well as the resultant nucleation and growth process. Mohite et al. reported a strong correlation between the aging time and film crystallinity [46]. The results showed that the crystallinity and grain size of perovskite films were significantly improved when the aging time of the precursor solution exceeds 24 h. Figure 3e shows the average grain size as a function of the solution of aging time. Along with the grain size growth, the XRD peak ratio of the MAPbI_3_/MAPbCl_3_ film increased to 2.73 when the solution aging time reached 24 h (Fig. 3f). The full width at half maximum (FWHM) of the peak of MAPbI_3_ at 14.28° (110) exhibits an increase in the crystalline quality as the aging time increases to a certain value. As the precursor solution ages, it gradually forms large seeds (or crystals). The precursor aging has also been proven to significantly affect the grain growth, phase purity, surface uniformity, and trap state density of the perovskite film. Meanwhile, adjusting the solvents and composition of the precursor has a vital role in the phase formation and crystalline properties of the perovskite films. A BA_2_MA_4_Pb_5_I_16_ film deposited using a *N*,*N*-dimethylformamide (DMF)/dimethyl sulfoxide (DMSO) mixed solvent showed a strong preferential orientation, in which the (111) diffraction intensity was about tenfold higher than that prepared with a DMF solvent (Fig. 3g) [17]. When the DMSO ratio increased, the FWHM of the (111) peak further decreased from 0.51° to 0.24° (Fig. 3h). Wang et al. used gamma-butyrolactone (GBL) and DMF co-solvents to deposit perovskite films. It was reported that the grain size and photovoltaic parameters of the device from the GBL solvent were significantly lower than those from the DMF solvent [55]. In addition, Iyer et al. regulated the perovskite crystallization and grain growth with DMSO as a Lewis base adduct [56].

#### Composition and Other Factors

The perovskite film morphology can be further tuned through the processing parameters, such as the substrate temperature, rotating speed, and thermal annealing. These processing parameters affect the solute diffusion and perovskite crystallization behavior through the thermal energy and centrifugal force. Thermal annealing promotes evaporation of the remaining organic residue. As shown in Fig. 3i, the main diffraction peaks of (n-BA)_2_(MA)_3_Pb_4_I_13_ and (iso-BA)_2_(MA)_3_Pb_4_I_13_ films at 14.10° and 28.37° represent the (111) and (202) crystallographic planes. Compared with the diffraction peak of the hot-casted (n-BA)_2_(MA)_3_Pb_4_I_13_ perovskite film, the diffraction intensities of (iso-BA)_2_(MA)_3_Pb_4_I_13_ were higher and narrower, which indicates that the branched-chain spacers improved the crystallization of the two-dimensional (2D) perovskites compared to the linear chain counterparts. In addition, the change in the diffraction intensity ratio between (111) and (202) of the hot-casted (iso-BA)_2_(MA)_3_Pb_4_I_13_ film implies that the crystal orientation of the film changed substantially [18]. Moon et al. used MAI and lead acetate (PbAc_2_) as precursors to form a high-quality perovskite layer [57]. The PbAc_2_ residue can be conveniently removed through by-product gas (3MAI + PbAc_2_ $\to$ MAPbI_3_ + 2MAAc) to accelerate the crystal growth to form a fully covered, pinhole-free, and highly crystalline perovskite film. Janssen et al. prepared a high-quality perovskite layer from a mixture of PbAc_2_, PbI, and MAI [22]. Huang et al. reported the use of methylammonium acetate (MAAc) as a general solvent to deposit high-quality perovskite films in an ambient environment. A constant substrate temperature (100 °C) was applied to promote solvent evaporation, resulting in supersaturation, and a rapid nucleation and crystal growth [58].

### Atmosphere

Different deposition conditions affect the crystallinity and surface morphology of as-cast perovskite films [59]. As one advantage of the hot-casting technology, the deposition is not sensitive to the processing environment; in other words, perovskite films can be deposited in ambient air, and the device shows excellent humidity stability [60–63]. Mori et al. prepared MAPbI_3_ films under ambient conditions (relative humidity = 42–48%) by combining a gas flow with hot-casting technology [64]. Owing to the difference in the centrifugal force between the center and edge of the substrate, the flowing gas can significantly accelerate the mass transfer and eliminate the nonuniformity of its thickness. Eslamian et al. prepared MAPbI_3_ perovskite films using a two-step sequential deposition method [65]. To achieve full coverage, a PbI_2_ solution with a low concentration was first sprayed onto a heated substrate as the substrate temperature neared the boiling point of the DMF solvent (153 °C). The deposition of thick PbI_2_ films by high-temperature spraying is more conducive to the deposition of thicker perovskite films than a conventional method applied at RT. Aguiar et al. found that in situ exposure to water vapor reduced or possibly eliminated the Cl^−^ retention in the FAPbI_3−*x*_Cl_*X*_ crystals [66]. During the treatment of FAPbI_3−*x*_Cl_*x*_, water vapor induced halide loss in the perovskite crystals. The chemical bonds between chlorine and its surrounding elements are extremely weak and can be easily broken by residual water vapor. Yang et al. further combined hot-casting technology with methylamine (MA) gas treatment to prepare dense and uniform perovskite films under high relative humidity [67]. Porous and rough MAPbI_3_ perovskite films prepared using hot casting can be transformed into dense and high-quality films with MA gas treatment [68]. In addition, Hao et al. treated MAPbI_3_ perovskite films with nondestructive ethanol/chlorobenzene, resulting in a coarsening of the perovskite grains and lateral grain growth of the MAPbI_3_ perovskite films [69]. Cheng et al. introduced a thermal radiation hot-casting method to solve the problems of temperature gradient and moisture intrusion during the deposition process [70]. A 500 W tungsten filament was used as the heat source. A smooth and dense perovskite film was then deposited by controlling the humidity and temperature gradients under ambient air.

### Single-Crystal Growth and Other Functional Layers

**Fig. 4:**
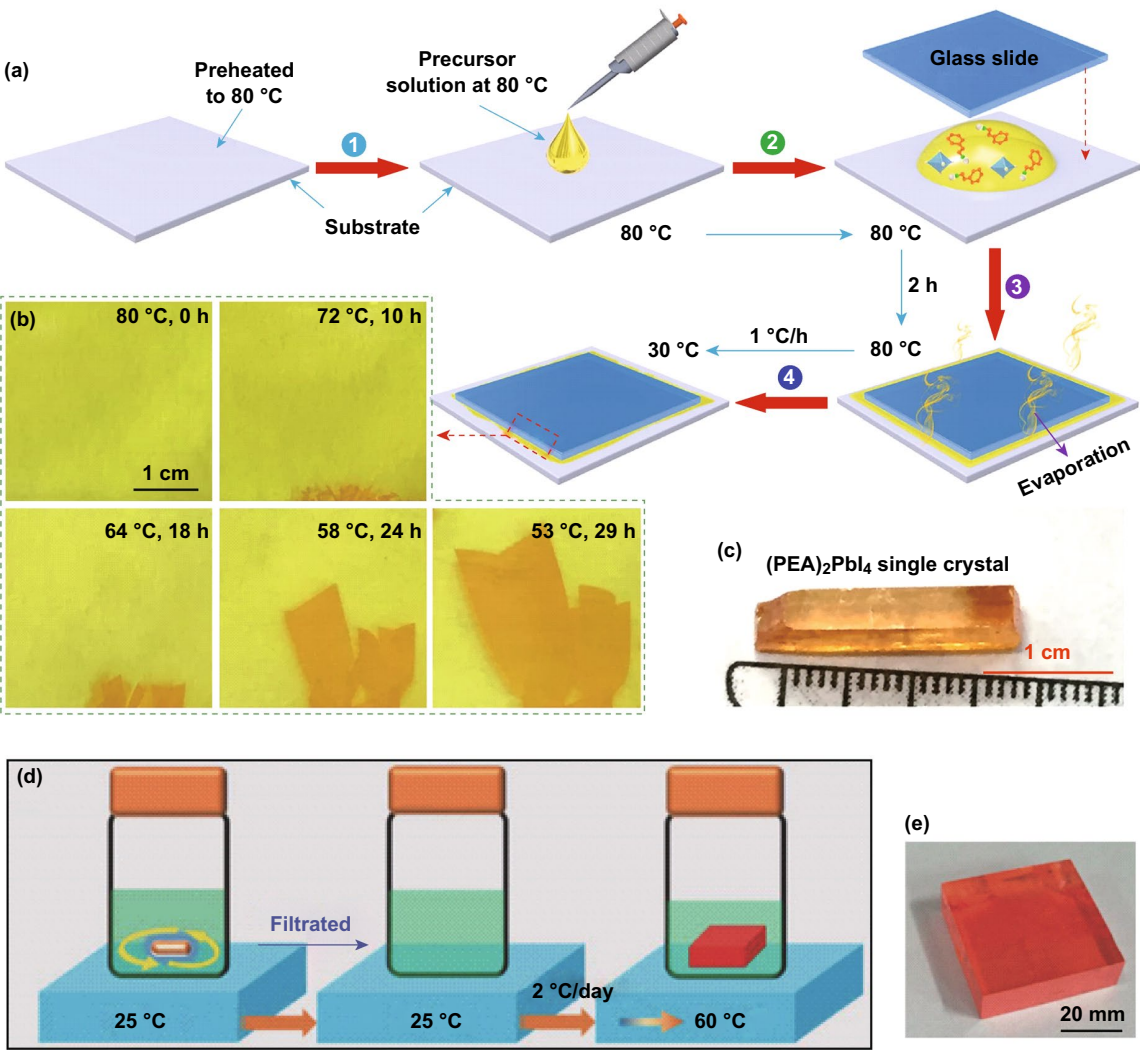
**a** Schematic illustration of the IPC procedure. **b** Photographs of PEA_2_PbI_4_ taken at different stages of the growth process. **c** Photograph of a typical PEA_2_PbI_4_ crystal [71]. Copyright © 2018 Elsevier, Ltd. **d** Schematic illustration of the low-temperature-gradient crystallization (LTGC) process for MAPbBr_3_ single crystals. **e** Photograph taken from a MAPbBr_3_ single crystal [72]. Copyright © 2018 WILEY-VCH Verlag GmbH & Co. KGaA, Weinheim

A single perovskite crystal exhibits advantages such as high light absorption, a long carrier lifetime, high carrier mobility, low trap state density, and excellent defect tolerance [71]. Liu et al. reported an induced peripheral crystallization (IPC) method to grow single crystals of multiple sizes (C_6_H_5_C_2_H_4_NH_3_)_2_PbI_4_ (PEA_2_PbI_4_). Figure 4a illustrates a schematic of the single-crystal growth process of PEA_2_PbI_4_. The hot precursor solution was transferred to a glass substrate at a constant of 80 °C. Slides are placed on droplets of the precursor solution, followed by squeezing, baking, and cooling. Figure 4b shows the growth of PEA_2_PbI_4_ crystals at different stages. At 80 °C, the solution is unsaturated, and thus it is impossible to form microcrystals. The solvent evaporated and the local concentration gradually increased. When the temperature decreased to 72 °C, small crystals formed on the edges of the glass slide. Figure 4c shows well-shaped single crystals with habitant rectangular a parallelepiped formation in a closed petri dish [71]. In addition, Liu et al. developed a low-temperature gradient crystallization method to grow a single crystal of CH_3_NH_3_PbBr_3_ with high carrier mobility by adjusting the crystal nucleation and growth, as shown in Fig. 4d, e [72].

A hot-casting technique can also be used to deposit other functional layers (such as SnO_2_, TiO_2_, NiO_*x*_, cuprous thiocyanate (CuSCN), or cuprous iodide (CuI)). For example, Lian et al. reported the hot-substrate deposition of poly(4-butylphenyl-diphenyl-amine) (PTPD) as an effective hole transport layer (HTL) and phenyl-C61-butyric acid methyl ester (PCBM) as an electron transport layer (ETL) [73]. The solvent volatilization caused by the hot substrate accelerates the self-organization of the PTPD, thereby improving the adhesion to the substrate with higher coverage and flatness. Hot-cast PCBM films show a higher uniformity and lower roughness, which helps reduce the current leakage and charge recombination. Hao et al. adjusted the deposition temperature of hot-cast NiO_*x*_ films to change the coordination structure and charge state of the NiO_*x*_. In addition, the hole injection efficiency at the NiO_*x*_/perovskite interface was improved, and the charge accumulation at the interface was effectively avoided [74].

## Advantages of Hot-Casting Technique

### Grain Size, Film Thickness, and Orientation

**Fig. 5:**
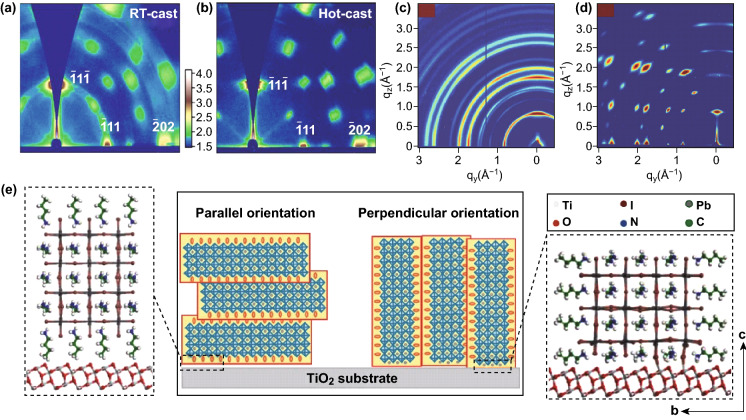
2D GIWAXS plots of BA_2_MA_3_Pb_4_I_13_ films processed using **a** RT casting and **b** hot casting [86]. Copyright © 2019 Elsevier Ltd. **c** GIWAXS data of the hot-cast films from DMF and **d** from the optimized 3:1 DMF: DMSO mixed solvent [17]. Copyright © 2017 WILEY-VCH Verlag GmbH & Co. KGaA, Weinheim. **e** Titanium oxide/(BA)_2_(MA)_3_Pb_4_I_13_ interface (optimized structures) for the parallel orientation (left) and perpendicular orientation (right) conditions [87]. Copyright © 2018 WILEY-VCH Verlag GmbH & Co. KGaA, Weinheim

A hot-casting technique has been widely applied to obtain relatively thick and preferentially orientated large-grain perovskite films. The larger grain size endows a reduction of the grain boundaries of the perovskite film. At the same time, there is a positive influence on the absorption, charge transport, and crystallinity of the perovskite films [75–77]. However, the increase in grain size may increase the density of the undesirable pinholes, resulting in direct contact between the HTL and ETL and the leakage current. The voids in the perovskite film will seriously damage the device performance [78–80]. In addition, the optoelectronic performance of the perovskite film is not directly related to the size of the crystalline domains [81]. A thick perovskite layer contributes to the harvesting of sufficient light absorption across the visible light range. In general, the concentration of the precursor solution and the rotation speed can be adjusted to tune the thickness of the perovskite layer. A thickness-insensitive device performance was observed when applying the hot-casting technique, which is important for the large-scale implementation of this deposition technology. When a long-chain organic cation layer is inserted into the inorganic framework to deviate the tolerance factor from a value of 1, the inorganic halide octahedrons are connected in a common apex and extend in a 2D direction to form 2D perovskites [82–85]. The insertion of an organic chain will hinder the charge extraction and collection. It is important to control the growth orientation of a 2D perovskite film to facilitate carrier transport. A preferentially orientated growth of 2D perovskites can be obtained through a hot-casting process. This enables the device to achieve an excellent carrier transferability and high PCE. Zhang et al. studied the crystal orientation of RT-cast and hot-cast BA_2_MA_3_Pb_4_I_13_ perovskite films using GIWAXS (Fig. 5a, b) [86]. The Debye–Scherrer diffraction rings of a BA_2_MA_3_Pb_4_I_13_ film at RT indicated a random orientation. Otherwise, the 2D perovskite film deposited using the hot-casting method exhibited sharp and discrete Bragg spots along the same ring, indicative of the preferred orientation of a BA_2_MA_3_Pb_4_I_13_ film. A hot-casted film with a strong vertical orientation provides a direct pathway for electron and hole extraction. In addition, it was further reported that the out-of-plane orientation was significantly enhanced in the hot-casted (iso-BA)_2_(MA)_3_Pb_4_I_13_ perovskite film by the sharp spots along *q*_z_ in GIWAXS [18]. Kanatzidis et al. reported that a mixed solvent (DMF:DMSO) can induce a better crystallinity and preferential orientation of the hot-casted BA_2_MA_4_Pb_5_I_16_ film (Fig. 5c, d) [17]. Compared with a pure DMF solvent, the DMF/DMSO mixed solvent dramatically increased the peak intensity of (111) and (202) by tenfold. As a possible reason for this, a small amount of polarized DMSO (owing to the S^δ+^ = O^δ−^ polarized bond) in the precursor solution can form strong hydrogen bonds with the organic cation, which helps change the crystallization rate and increase the film crystallinity. This improves the carrier transport by controlling the degree of preferential orientation of the perovskite film. According to the density functional theory (DFT), the interface between the dimensional-reduced perovskite nuclei and the TiO_2_ substrate was calculated and analyzed (Fig. 5e) [87]. BA- and MA-attached TiO_2_ substrates represent parallel and perpendicular orientations, respectively. After the geometry optimization, the interfacial energy of parallel and perpendicular orientated (BA)_2_(MA)_3_Pb_4_I_13_/TiO_2_ is 0.5 and 8.9 eV, respectively. It is inferred that when a (BA)_2_(MA)_3_Pb_4_I_13_ nucleus is formed on a TiO_2_ substrate, perpendicular orientations are highly thermodynamically preferred.

### Defects and Recombination

**Fig. 6:**
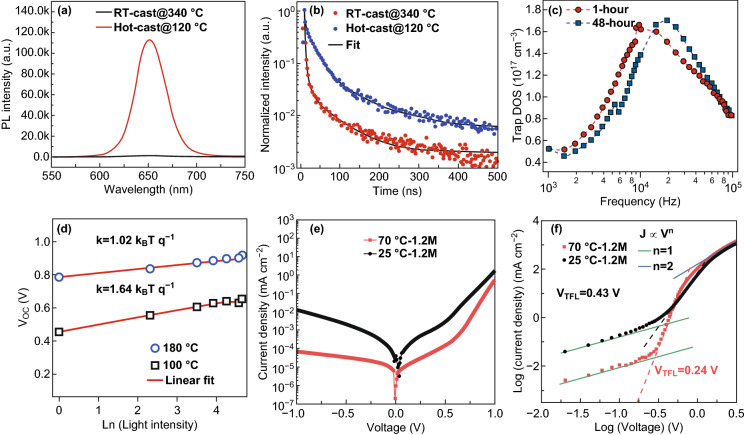
**a**, **b** PL and TRPL spectra of CsPbI_2_Br films fabricated using RT and hot casting [88]. Copyright © 2019 The Royal Society of Chemistry. **c** Trap density of states for the device with 1-h and 48-h aged perovskite precursors [46]. Copyright © 2017 WILEY-VCH Verlag GmbH & Co. KGaA, Weinheim. **d** *V*_oc_ as a function of illumination light intensity for a large-grain device (180 °C) and a small-grain device (100 °C) [19]. Copyright © 2017 American Association for the Advancement of Science. **e** *J*–*V* characteristics of PSCs swept from 1.0 to − 1.0 V in the dark. **f** *J*–*V* characteristics of hole-only devices [53]. Copyright © 2016 WILEY-VCH Verlag GmbH & Co. KGaA, Weinheim

A nonradiative recombination deteriorates the PSC performance through the following pathways. The electrons fall back to the valence band, resulting in radiative recombination. Holes (electrons) are transferred back to the perovskite layer, resulting in minority recombination at the interface. The perovskite layer with pores will then cause direct contact with the functional layers, thereby generating carrier recombination. Numerous defect states in the device form recombination centers to capture the carriers, causing a nonradiative recombination process. Some deep-level traps are the main pathway for a carrier loss. In addition, the nonradiative recombination of carriers inside the device also directly affects the achievable *V*_oc_ of the PSCs. Therefore, the deposition of low-defective perovskite thin films is important for suppressing nonradiative recombination. For hot-casted perovskite films, large grain sizes help reduce the number of grain boundaries to suppress the charge trapping. PL and TRPL have been widely used to study the charge carrier kinetics of perovskite films deposited using conventional RT-casting and a hot-casting technique. As shown in Fig. 6a, b, the PL intensity and carrier lifetime of the hot-cast perovskite film are much higher than that of the RT-cast film, indicating a significant decrease in the nonradiative recombination rate in the hot-cast CsPbI_2_Br film. Figure 6c depicts the calculated density of state (DOS) from the impedance spectroscopy for 1- and 48-h aged devices, respectively. Both DOS values fluctuate at approximately 1.7 × 10^17^ cm^−3^. The 48-h aging device tends to make trap state peaks toward lower frequencies, indicative of shallow trap energy and low-frequency dielectric loss related to the reduced grain boundaries in the perovskite film. Figure 6d shows the dependence of the measured *V*_oc_ as a function of incident light intensity for a large-grain device (180 °C) and a small-grain device (100 °C). The linear-fitting slope of the small-grain device was 1.64 *k*_B_*T*/*q*, which was significantly higher than that of a large-grain device (1.02 *k*_B_*T*/*q*). This difference indicates a more severe trap-assisted recombination in the device with smaller grain size. Meanwhile, hot-casted devices have higher rectification coefficients and less leakage current in the dark current curves (Fig. 6e), which means smaller recombination compared to the RT-casted device. Figure 6f shows the *J*–*V* characteristics of hole-only devices with an ITO/NiO_*x*_/perovskite/PTAA/MoO_3_/Ag configuration to estimate the hole mobility and defect density of perovskite films deposited at different temperatures. The defect densities of devices fabricated at 70 and 25 °C were derived as 1.00 × 10^15^ cm^−3^ and 7.50 × 10^15^ cm^−3^, respectively. This was mainly due to the increase in grain size and the boundary reduction of devices deposited at 70 °C.

### Efficiency and Stability

#### Device Performance

The PCE, stability, and cost are three main limiting factors for the practical application of PSCs. Table 1 summarizes the recent advances of PCEs when using a hot-casting technique. MAPbI_3_ and FAPbI_3_ are the two dominant light harvesters in PSCs, with energy bandgaps of 1.55 and 1.47 eV and a theoretical maximum PCE of 31.3% and 32.5%, respectively [89, 90]. Nie et al. first reported the fabrication of a millimeter grain size of perovskite films, enabling a PCE of 17.48% with negligible hysteresis [19]. Marks et al. reported a hot-casting process to control the Cl^−^ incorporation and achieved a PCE of 18.2% for a small area (0.09 cm^2^) and 15.4% for a large area (1 cm^2^) [11]. Chen et al. reported the deposition of an 850-nm-thick perovskite film together with a PCE of up to 19.54% [53]. Tsai et al. reported a 12.52% PCE without hysteresis based on BA_2_MA_3_Pb_4_I_13_ 2D perovskite [91]. We believe that the PCE of PSCs using a hot-casting technology can catch up to that using a solution method in the future. Meanwhile, the improvement in the long-term stability for PSCs is closely related to the perovskite composition [17, 24, 92], device structure [93], and encapsulation [11].

**Table 1:** Summary of photovoltaic parameters for PSCs with hot-casting technique

Perovskites	Device structure	*V*_oc_ (V)	*J*_sc_ (mA cm^−2^)	FF	PCE (%)	Refs.
MAPbI_3−*x*_Cl_*x*_	FTO/PEDOT:PSS/perovskite/PCBM/Al	0.94	22.40	0.83	17.48	[19]
MAPbI_3_	FTO/NiO_*x*_/perovskite/PCBM/Ag	1.11	21.14	0.80	18.84	[94]
MAPbI_3−*x*_Cl_*x*_	FTO/SnO_2_/perovskite/spiro-OMeTAD/Au	1.12	22.50	0.76	19.10	[70]
MAPbI_3−*x*_Cl_*x*_	FTO/c-TiO_2_/perovskite/PTAA/Au	1.10	21.40	0.78	18.30	[95]
MA_0.6_FA_0.38_Cs_0.02_PbI_2.975_Br_0.025_	ITO/PTAA/perovskite/C_60_/BCP/Cu	1.09	23.10	0.77	19.50	[96]
MAPbI_3_	FTO/c-TiO_2_/perovskite/spiro-OMeTAD/Ag	1.00	20.60	0.64	13.1	[37]
MAPbI_3−*x*_CI_*x*_	ITO/PEDOT:PSS/perovskite/PCBM/LiF/Al	1.00	18.60	0.75	14.6	[22]
MAPbI_3−*x*_CI_*x*_	FTO/NiO_*X*_/perovskite/PCBM/PEI/Ag	1.08	21.2	0.79	18.2	[11]
MAPbI_3−*x*−*y*_Br_*x*_CI_*y*_	ITO/PEDOT:PSS/perovskite/PC_61_BM/Ca/Al	1.10	19.25	0.78	16.52	[51]
FA_0.25_MA_0.75_PbI_3_	FTO/c-TiO_2_/perovskite/spiro-OMeTAD/Au	0.97	22.8	0.66	14.6	[54]
MAPbI_3−*x*_CI_*x*_	ITO/PEDOT:PSS/perovskite/PCBM/Al	0.94	20.92	0.77	15.22	[46]
MAPbI_3_	ITO/NiO_*X*_/MAPbI_3_/PCBM/BCP/Ag	1.11	22.70	0.78	19.54	[53]
MAPbI_3_	FTO/c-TiO_2_/m-TiO_2_/perovskite/spiro-OMeTAD/Au	1.07	21.32	0.70	16.01	[56]
MAPbI_3_	ITO/PEDOT:PSS/perovskite/PCBM/Ag	0.92	21.96	0.70	14.24	[67]
FAPbI_3−*x*_CI_*x*_	FTO/c-TiO_2_/perovskite/spiro-OMeTAD/Ag	0.96	18.93	0.66	12.07	[66]
MAPbI_3_	FTO/c-TiO_2_/perovskite/spiro-OMeTAD/Au	1.01	21.80	0.74	16.32	[64]
MAPbI_3_	FTO/c-TiO_2_/perovskite/spiro-OMeTAD/Au	1.07	22.67	0.77	18.74	[97]
CsPbI_2_Br	ITO/SnO_2_/perovskite/PTAA/MoO_3_/Al	1.19	14.54	0.74	13.80	[88]
Ag_2_BiI_5_	FTO/c-TiO_2_/m-TiO_2_/perovskite/PTAA/Au	0.69	6.04	0.62	2.60	[92]
BA_2_MA_4_Pb_5_I_16_	ITO/PEDOT:PSS/perovskite/PCBM/Al	1.00	11.44	0.75	8.71	[23]
BA_2_MA_3_Pb_4_I_13_	FTO/c-TiO_2_/m-TiO_2_/perovskite/spiro-OMeTAD/Au	0.97	17.19	0.53	9.03	[98]
BA_2_MA_3_Pb_4_I_13_	FTO/PEDOT:PSS/perovskite/PCBM/Al	1.01	16.76	0.74	12.52	[91]
BA_2_MA_3_Pb_4_I_13_	ITO/PTAA/layer perovskite/PCBM/BCP/Cu	1.13	18.90	0.59	12.7	[24]
(CPEA)_2_MA_4_Pb_5_I_16_	FTO/c-TiO_2_/perovskite/spiro-OMeTAD/Au	0.99	19.92	0.60	11.86	[99]
BA_2_MA_3_Pb_4_I_13_	FTO/c-TiO_2_/perovskite/spiro-OMeTAD/Au	1.08	19.45	0.58	12.17	[87]
MA_2_PbI_4_	FTO/c-TiO_2_/perovskite/spiro-OMeTAD/Au	1.06	21.00	0.76	16.92	[100]
(GA)(MA)_3_Pb_3_I_10_	FTO/c-TiO_2_/perovskite/spiro-OMeTAD/Au	1.00	20.70	0.66	13.87	[101]

# FTO, fluorine-doped tin oxide; ITO, indium-tin-oxide; SnO_2_, tin oxide; TiO_2_, titanium dioxide; PCBM, phenyl C61 butyric acid methyl ester; Al, aluminum; Ag, silver; Au, gold; PTAA, poly[bis(4-phenyl)(2,4,6-trimethylphenyl)aMine]; LiF, lithium fluoride; spiro-OMeTAD, 2,2′,7,7′-tetrakis(*N*,*N*-di-*p*-methoxyphenylamine)-9,9′-spirobifluorene; CPEA, ClC_6_H_4_C_2_H_4_NH_3_^+^; GA, guanidinium

#### Stability

During the film deposition, device testing and storage, both water and oxygen directly affect the PSC performance and stability [102–104]. Taking MAPbI_3_ as an example, first, water vapor can dissolve the perovskite material (3), and MAI is then decomposed to form a mixture of MA and HI (4); however, HI will either react with O_2_ to form H_2_O and I_2_ (5), or self-decompose (6). In general, MAPbI_3_ continues to decompose after exposure to moisture.

3 $${\text{CH}}_{3} {\text{NH}}_{3} {\text{PbI}}_{3} ( {\text{s)}} \mathop \leftrightarrow \limits^{{{\text{H}}_{2} {\text{O}}}} {\text{CH}}_{3} {\text{NH}}_{3} {\text{I(aq)}} + {\text{PbI}}_{2} ( {\text{s)}}$$

4 $${\text{CH}}_{3} {\text{NH}}_{3} {\text{I(aq)}} \leftrightarrow {\text{CH}}_{3} {\text{NH}}_{2} ( {\text{aq)}} + {\text{HI(aq)}}$$

5 $$4{\text{HI }}({\text{aq}}) + {\text{O}}_{2} ({\text{g}}) \leftrightarrow 2{\text{I}}_{2} ( {\text{s)}} + 2{\text{H}}_{2} {\text{Oi}}$$

6 $$2{\text{HI(aq)}}\mathop \leftrightarrow \limits^{hv} {\text{H}}_{2} {\text{g}} + {\text{I}}_{2} ( {\text{s)}} .$$

**Fig. 7:**
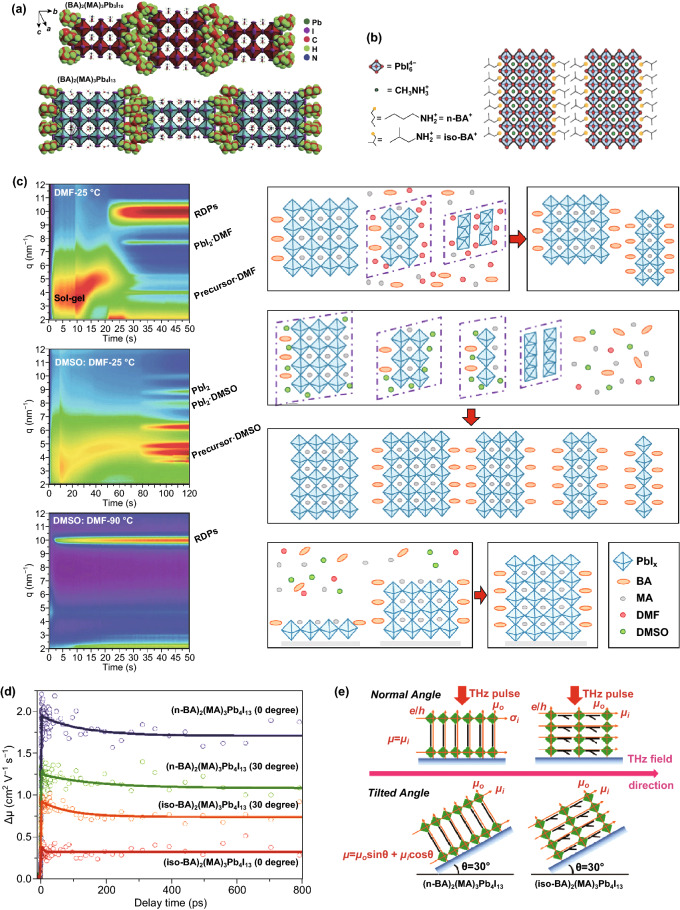
Schematic illustration of perovskite crystal structures: **a** (BA)_2_(MA)_3_Pb_4_I_13_ and (BA)_2_(MA)_3_Pb_4_I_13_; **b** (iso-BA)_2_(MA)_3_Pb_4_I_13_. Copyright © 2017 WILEY-VCH Verlag GmbH & Co. KGaA, Weinheim. **c** In situ GIWAXS measurements and schematic models [87]. Copyright © 2018 WILEY-VCH Verlag GmbH & Co. KGaA, Weinheim. **d** The photoconductivity kinetics from TRTS for RT (n-BA)_2_(MA)_3_Pb_4_I_13_ and (iso-BA)_2_(MA)_3_Pb_4_I_13_ films excited at 400 nm under an excitation density of 3.4 × 10^13^ ph cm^−2^ pulse^−1^ with a different pump-probe geometry at both normal positions. **e** Schematics of the composition of the charge mobility detected by TRTS with different incident angles of THz probe pulse for two samples. Copyright © 2017 WILEY-VCH Verlag GmbH & Co. KGaA, Weinheim

The poor stability of a PSC device severely limits its commercialization. Adjusting the ABX_3_ perovskite composition and/or increasing the crystalline quality is key in improving long-term stability. In this regard, long-chain organic cations (^+^NH_3_-R-NH_3_^+^ or R-NH_3_^+^) have been widely introduced as a substitute to a site cations. These 2D perovskites exhibit excellent long-term stability [62, 91], in which the organic layer and the inorganic layer alternately form a layered structure. The larger organic cations in the 2D perovskite crystal structure improve the humidity stability owing to the hydrophobic nature of long-chain organic cations. Pure 2D PSCs exhibit good stability but low PCE [17, 23]. Interestingly, excellent optoelectronic properties and stability can be achieved by combining 3D perovskite and 2D Ruddlesden–Popper perovskite [105, 106]. To successfully achieve this 3D/2D structure, it is necessary to control the growth direction allowing the carriers to be transported across the plane; otherwise, the transmission of photogenerated charge carriers will be inhibited by the long organic chain. Perovskite films grown perpendicular to the substrate direction can be obtained using hot-casting techniques. This allows the device to achieve an excellent carrier transportability and high PCE (Fig. 7a, b). Kanatzidis et al. conducted stability tests for BA_2_MA_4_Pb_5_I_16_ films in ambient air (RH ≈ 30%). A BA_2_MA_4_Pb_5_I_16_ film exhibits excellent environmental stability [17]. The 2D perovskite deposited using a hot-casting technique can maintain its inherent performance for a long timeframe, indicating superior stability under humid and other environmental conditions [18]. Figure 7c schematically illustrates that a hot substrate can promote a rapid transition from a disordered precursor solution to a perovskite phase without the growth of an intermediate phase, thereby obtaining a low-dimensional perovskite film with a vertical orientation and a high phase purity [87]. The carrier transport dynamics of 2D perovskites have been recognized to be related to the differences in the crystal orientation. Liang et al. used time-resolved terahertz (THz) spectroscopy (TRTS) to measure the photoconductivity kinetics of n-BA and iso-BA-based 2D perovskites from the transient charge carrier mobility (Δ*µ*), in-plane mobility (*µ*_i_), and out-of-plane mobility (*µ*_o_). A THz probe beam is perpendicular to the (n-BA)_2_(MA)_3_Pb_4_I_13_ and (iso-BA)_2_(MA)_3_Pb_4_I_13_ films (Fig. 7d). The measured carrier mobilities were 1.88 and 0.38 cm^2^ V^−1^ s^−1^ at 2 ps, respectively. The value of *µ*_o_ of (iso-BA)_2_(MA)_3_Pb_4_I_13_ (1.35 cm^2^ V^−1^ s^−1^) was much higher than that of (n-BA)_2_(MA)_3_Pb_4_I_13_. The (iso-BA)_2_(MA)_3_Pb_4_I_13_ perovskite film exhibits a strong crystalline orientation, which increases charge mobility [18].

All inorganic perovskites are also a promising choice for improving the long-term stability, which have better thermal stability owing to the presence of an inorganic cation with a higher thermal decomposition temperature. Zheng et al. adopted hot casting to deposit an inorganic CsPbI_2_Br perovskite film at low temperatures [88]. The phase transition temperature of CsPbI_2_Br was reduced to 120 °C, and a dense and pinhole-free CsPbI_2_Br perovskite film with a large grain size was formed. An ultrahigh substrate temperature is not necessary for the hot-casting process. Zhao et al. evaluated the thermodynamic stability of the materials through DFT calculations [107]. The positive decomposition enthalpy ($\Delta H_{\text{dec}}$) of CsPbBr_3_ indicates high thermodynamic stability. This is different from that of CsPbI_3_, CsPbI_2_Br, and CsPbBr_2_I with a negative $\Delta H_{\text{dec}}$. Song et al. deposited a uniform large-area cesium-based 2D perovskite thin film using hot-casting technology. Compared with an RT-casting film, a hot-casting film has a higher PL strength and longer carrier life [108]. Mathews et al. deposited silver bismuth iodide (AgBiI_4_ and Ag_2_BiI_5_) through dynamic hot casting under an ambient atmosphere [92]. The hot-casting technique facilitates the deposition of a needle-free uniform film with a large grain size by increasing the solubility of AgI.

### Upscaling Deposition

**Fig. 8:**
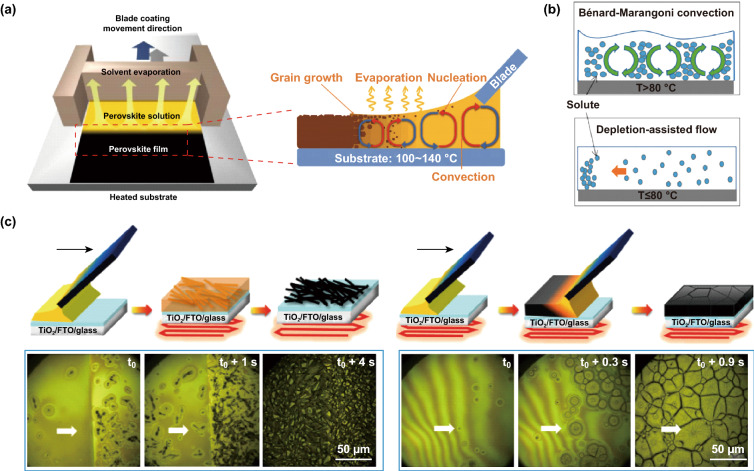
**a** Scheme of the doctor-blade coating method [96]. Copyright © 2017 WILEY-VCH Verlag GmbH & Co. KGaA, Weinheim. **b** Schematic illustration of the Bénard–Marangoni convection and depletion-assisted flow of CsPbI_2_Br fluid at higher and lower processing temperatures, respectively [107]. Copyright © 2019 Elsevier Inc. **c** Schematic of the process and real-time optical microscopy: DMSO:GBL-blade-25 °C film and DMSO:GBL-blade-150 °C film during thermal annealing [97]. Copyright © 2018 Elsevier Inc.

As a solution-processable photovoltaic technology, hot casting is mostly applied to small area devices (< 1 cm^2^) in the laboratory. Based on the rapid development and advantages of hot-casting technology, integration with other scalable deposition technologies is a promising way to achieve upscaled manufacturing of PSCs. The basic idea of hot-casting technology is to stabilize the temperature of the substrate and precursor solution concurrently, thereby affecting the nucleation rate and film morphology through a rapid supersaturation of the precursor solution. Coincidentally, the substrate temperature has been reported to have an important effect on the film morphology in blade coating, spray coating, and inkjet printing. Figure 8a schematically illustrates the blade coating deposition on a hot substrate [96, 109–111]. Huang et al. introduced a small amount of Cs^+^ cations and Br^−^ anions into the perovskite precursor solution to obtain a MA_0.6_FA_0.38_Cs_0.02_PbI_2.975_Br_0.025_ film and improve the phase purity [96]. The rapid film-forming process of the hot-casting technique may be effective in suppressing the “solution flow” during the dry film stage. Bénard–Marangoni convection caused by the thermal gradient cannot be ignored in a CsPbI_2_Br fluid, which will pull the accumulated solutes in an area with the highest surface tension and inhibit the formation of a continuous film (Fig. 8b) [107]. At the optimal processing temperature of 80 °C, a large-area, high-crystallinity, uniform, and pinhole-free CsPbI_2_Br film can be obtained. The slow solvent removal and the presence of intermediate phases during the thermal annealing result in poor film coverage and low crystalline quality of the RT-casted film. The hot casting at 150 °C induces a direct crystal growth, which allows the formation of a compact, pinhole-free, and uniform film (Fig. 8c) [97].

**Fig. 9:**
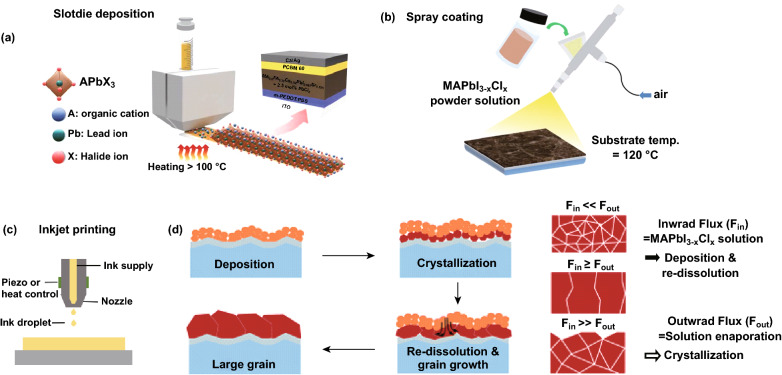
Common scalable solution deposition methods. **a** Slot-die deposition [112]. Copyright © 2019 WILEY-VCH Verlag GmbH & Co. KGaA, Weinheim. **b** Spray coating [95]. **c** inkjet printing. **d** Crystalline grain growth and the morphology of the formed crystalline grains in the perovskite film with respect to the balance between *F*_in_ and *F*_out_ [95]. Copyright © 2016 The Royal Society of Chemistry

Spray coating and inkjet printing have also been applied to accelerate the evaporation of solvent molecules by increasing the substrate temperature and finally obtain a uniform and dense perovskite film. Meanwhile, slot-die deposition [112], spray coating [65, 95], and inkjet printing are low-cost, high-throughput, and substrate-compatible deposition thin-film technologies (Fig. 9a–c). Perovskite crystal grains can grow into larger crystals through re-dissolution, grain-merging, and re-crystallization. The balance of an inward flux (*F*_in_) and an outward flux (*F*_out_) can be controlled through deposition parameters such as the solution concentration, solvent mixture, and substrate temperature (Fig. 9d) [95]. Owing to the high sensitivity of perovskite materials in the atmospheric environment, the development of these technologies has been limited. When integrated with hot-casting technology, the sensitivity of the perovskite materials can be improved through the rapid film formation and introduction of all-inorganic components and long alkyl amine chains.

## Conclusion and Prospects

The quality of a perovskite film is a key factor in determining the ultimate performance of PSCs. Hot-casting technology shows excellent device performance and commercialization prospects in the deposition of high-quality perovskite films. The current research status of this technology was comprehensively summarized and discussed in this review. Based on the classical LaMer model, the effects of various depositing parameters on different stages of nucleation and growth of perovskite films were analyzed. The results show that the adjustment of the film formation parameters such as the substrate temperature, thermal annealing, precursor chemistry, and experimental environment can improve the nucleation and crystallization of perovskite, as well as obtain a higher-quality perovskite film and PSCs with superior performance. In addition, the review highlighted the advantages of hot-casting techniques for the deposition of perovskite films, such as large grain sizes, insensitive thicknesses, and preferred orientations of low-dimensional perovskites. From the perspective of stability, the application of hot-casting technology in inorganic, lead-free, and low-dimensional perovskite films was also summarized. Finally, the prospects of an upscaling of hot-casting technology or integration with other scalable deposition technologies were discussed.

However, there are still some remaining questions about this promising technology that has yet to be fully answered. For example, the chemical reaction of the precursors and the crystallization kinetics process has not been fully disclosed. The interface mechanism of a hot-cast film in contact with a functional layer has not been clearly explained, and the PCE of a device prepared using a hot-casting technique is still lower than that from a conventional solution deposition. The mechanism of water invasion toward hot-cast perovskite surfaces and related stability issues has not been reported in detail in the literature. According to the particular film-forming process and film properties of the hot-casting technology, the following issues should be taken into consideration for the maturity of this technology: The concurrent solvent evaporation and perovskite crystallization over an extremely short period of time (approximately 3–5 s) makes the control of the crystallization a challenge. Adjusting the solvent composition, additives, and solvent engineering will play a role in the proper control of the crystallization. In addition, from a more microscopic perspective, the temperature distribution around the crystal nucleus, grain boundaries, and unit cells during nucleation growth should be analyzed. The mechanism of thermal energy at each stage and the effect of the release of residual thermal energy on the film should be interpreted from a thermodynamic perspective. The integration of a hot-casting technology with other scalable deposition technologies (such as blade coating, spray coating, inkjet printing, screen printing, and slot-die deposition) in the deposition of high-quality perovskite films with a large area, high throughput, and low cost should be highly desirable for future industrial application of this promising technique. Moreover, hot-casting technology has extremely broad prospects in the growth of low-dimensional perovskite films, inorganic perovskite films, and single-crystal growth. In addition, hot-casting technology can be used as an early exploration tool for new materials and structures. This is of great significance for the rapid screening of early research directions. Owing to the rapid and controlled crystallization and short film-forming process, we look forward to wide implementation of a hot-casting technique applied to the upscaling deposition of perovskite films and related optoelectronic devices in the near future.
